# 4-Ethoxy­anilinium hexa­fluoro­phosphate monohydrate

**DOI:** 10.1107/S1600536810018404

**Published:** 2010-05-26

**Authors:** Xue-qun Fu

**Affiliations:** a Ordered Matter Science Research Center, Southeast UniVersity, Nanjing 210096, People’s Republic of China

## Abstract

In the crystal of the title compound, C_8_H_12_NO^+^·PF_6_^−^·H_2_O, inter­molecular N—H⋯F, N—H⋯O and O—H⋯F hydrogen bonds link the mol­ecules into chains along the *c* axis and C—H⋯π contacts further stabilize the structure. The F atoms of one of the hexa­fluoro­phosphate anions are disordered over two sets of sites with site-occupancy factors of 0.27 (3) and 0.73 (3).

## Related literature

For related structures, see: Fu (2009*a* ▶,*b* ▶). The title compound was studied as part of our search for ferroelectric compounds, which usually have a phase transition.For background to phase transition materials, see: Li *et al.* (2008 ▶); Zhang *et al.* (2009 ▶).
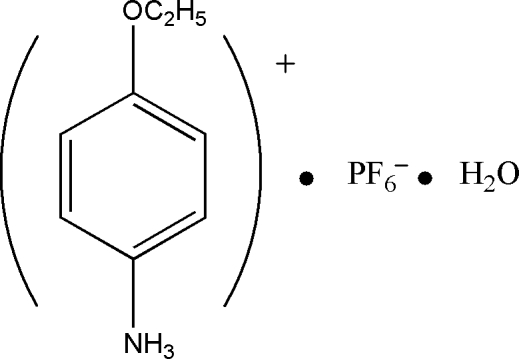


## Experimental

### 

#### Crystal data

C_8_H_12_NO^+^·PF_6_^−^·H_2_O
*M* *_r_* = 301.17
Monoclinic, 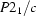

*a* = 17.498 (4) Å
*b* = 5.1236 (10) Å
*c* = 14.793 (3) Å
β = 111.68 (3)°
*V* = 1232.4 (4) Å^3^
*Z* = 4
Mo *K*α radiation
μ = 0.29 mm^−1^
*T* = 298 K
0.4 × 0.3 × 0.2 mm

#### Data collection

Rigaku SCXmini diffractometer
Absorption correction: multi-scan (*CrystalClear*; Rigaku, 2005 ▶) *T*_min_ = 0.900, *T*_max_ = 0.943
12124 measured reflections
2820 independent reflections
1890 reflections with *I* > 2σ(*I*)
*R*_int_ = 0.054

#### Refinement

*R*[*F* ^2^ > 2σ(*F* ^2^)] = 0.059
*wR*(*F* ^2^) = 0.164
*S* = 1.05
2820 reflections
226 parameters
235 restraints
H atoms treated by a mixture of independent and constrained refinement
Δρ_max_ = 0.45 e Å^−3^
Δρ_min_ = −0.48 e Å^−3^

### 

Data collection: *CrystalClear* (Rigaku, 2005 ▶); cell refinement: *CrystalClear*; data reduction: *CrystalClear*; program(s) used to solve structure: *SHELXS97* (Sheldrick, 2008 ▶); program(s) used to refine structure: *SHELXL97* (Sheldrick, 2008 ▶); molecular graphics: *SHELXTL* (Sheldrick, 2008 ▶); software used to prepare material for publication: *PRPKAPPA* (Ferguson, 1999 ▶).

## Supplementary Material

Crystal structure: contains datablocks I, New_Global_Publ_Block. DOI: 10.1107/S1600536810018404/zq2040sup1.cif

Structure factors: contains datablocks I. DOI: 10.1107/S1600536810018404/zq2040Isup2.hkl

Additional supplementary materials:  crystallographic information; 3D view; checkCIF report

## Figures and Tables

**Table 1:** Hydrogen-bond geometry (Å, °) *Cg*1 is the centroid of the C1–C6 ring.

*D*—H⋯*A*	*D*—H	H⋯*A*	*D*⋯*A*	*D*—H⋯*A*
N1—H1*A*⋯F2′	0.89	2.16	3.03 (2)	167
N1—H1*A*⋯F2	0.89	2.17	3.049 (10)	169
N1—H1*B*⋯F1^i^	0.89	2.22	3.066 (8)	158
N1—H1*B*⋯F1′^i^	0.89	2.28	3.09 (3)	152
N1—H1*C*⋯O1*W*^ii^	0.89	2.22	2.883 (3)	131
N1—H1*C*⋯F3^ii^	0.89	2.46	3.137 (12)	133
N1—H1*C*⋯F3′^ii^	0.89	2.40	3.01 (3)	126
N1—H1*C*⋯F6′^ii^	0.89	2.51	2.96 (2)	112
O1*W*—H1*WB*⋯F3′^iii^	0.82 (1)	2.20 (3)	2.97 (3)	156 (4)
O1*W*—H1*WB*⋯F3^iii^	0.82 (1)	2.28 (3)	3.030 (12)	152 (4)
O1*W*—H1*WA*⋯F4^i^	0.82 (3)	2.24 (2)	3.040 (12)	164 (4)
O1*W*—H1*WA*⋯F4′^i^	0.82 (3)	2.11 (3)	2.88 (2)	155 (4)
O1*W*—H1*WA*⋯F5^i^	0.82 (3)	2.48 (4)	2.951 (16)	117 (3)
C8—H8*C*⋯*Cg*1^iv^	0.96	3.16	4.023 (5)	150

Symmetry codes: (i) 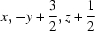; (ii) 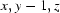; (iii) 
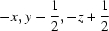
; (iv) 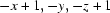.

**Fig. 1.**
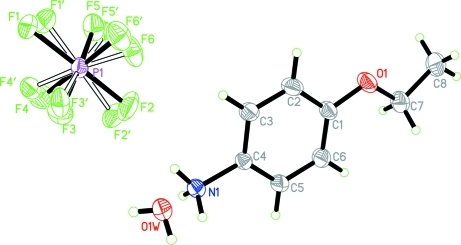
The molecular structure of the title compound with the atomic numbering scheme. Displacement ellipsoids are drawn at the 30% probability level, and all H atoms have been omitted for clarity.

**Fig. 2.**
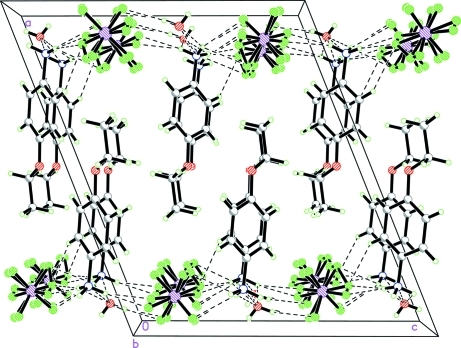
A view of the packing of the title compound, stacking along the c axis. Dashed lines indicate hydrogen bonds.

## supplementary crystallographic information

### Comment

As a continuation of our study of dielectric-ferroelectric materials, including organic ligands (Li *et al.*, 2008), metal-organic coordination compounds (Zhang *et al.*, 2009), and organic-inorganic hybrids, we studied the dielectric properties of the title compound. Unfortunately, there was no distinct anomaly observed from 93 to 350 K. In this article, the crystal structure of this compound is presented. The crystal structures of 4-ethoxyanilinium together with other anions are known (Fu, 2009a,b).

The asymmetric unit of the crystal structure consists of one almost coplanar protonated 4-ethoxyanilinium cation with the C2—C1—O1—C7 and C1—O1—C7—C8 torsion angles of 172.9 (3) and 177.4 (3)°, respectively, one hexafluorophosphate anion for which the F atoms are disordered over two sets of positions with site-occupancy factors of 0.27 (3) and 0.73 (3), and one water molecule (Fig.1). In the crystal structure, several intermolecular N—H···F and O—H···F hydrogen bonds link all species to chains along the c axis (Fig.2). In addition, C—H···π interactions further stabilize the crystal structure.

### Experimental

1.37 g (10 mmol) of 4-Ethoxybenzenamine was firstly dissolved in 50 ml ethanol, to which hexafluorophosphoric acid (70%, w/w) was then added until the solution becomes acidic under stirring. Single crystals of the title compond were prepared by slow evaporation at room temperature of the acidic solution after 3 days.

### Refinement

The water H atoms were found in Fourier difference maps and were refined freely. Positional parameters of all other H atoms were calculated geometrically and allowed to ride on the C and N atoms to which they are bonded, with U_iso_(H) = 1.2U_eq_(C,N). The F atoms of the hexafluorophosphate anion are disordered in two orientations with site-occupancy factors of 0.73 (3) and 0.27 (3).

### Crystal data

**Table:**

C_8_H_12_NO^+^·PF_6_^−^·H_2_O	*F*(000) = 616
*M**_r_* = 301.17	*D*_x_ = 1.623 Mg m^−^^3^
Monoclinic, *P*2_1_/*c*	Mo *K*α radiation, λ = 0.71073 Å
Hall symbol: -P 2ybc	Cell parameters from 4774 reflections
*a* = 17.498 (4) Å	θ = 3.1–27.7°
*b* = 5.1236 (10) Å	µ = 0.29 mm^−^^1^
*c* = 14.793 (3) Å	*T* = 298 K
β = 111.68 (3)°	Prism, colourless
*V* = 1232.4 (4) Å^3^	0.4 × 0.3 × 0.2 mm
*Z* = 4	

### Data collection

**Table:**

Rigaku SCXmini diffractometer	2820 independent reflections
Radiation source: fine-focus sealed tube	1890 reflections with *I* > 2σ(*I*)
graphite	*R*_int_ = 0.054
Detector resolution: 13.6612 pixels mm^-1^	θ_max_ = 27.5°, θ_min_ = 3.1°
ω scans	*h* = −22→22
Absorption correction: multi-scan (*CrystalClear*; Rigaku, 2005)	*k* = −6→6
*T*_min_ = 0.900, *T*_max_ = 0.943	*l* = −19→19
12124 measured reflections	

### Refinement

**Table:**

Refinement on *F*^2^	Primary atom site location: structure-invariant direct methods
Least-squares matrix: full	Secondary atom site location: difference Fourier map
*R*[*F*^2^ > 2σ(*F*^2^)] = 0.059	Hydrogen site location: inferred from neighbouring sites
*wR*(*F*^2^) = 0.164	H atoms treated by a mixture of independent and constrained refinement
*S* = 1.05	*w* = 1/[σ^2^(*F*_o_^2^) + (0.0764*P*)^2^ + 0.6523*P*] where *P* = (*F*_o_^2^ + 2*F*_c_^2^)/3
2820 reflections	(Δ/σ)_max_ = 0.003
226 parameters	Δρ_max_ = 0.45 e Å^−^^3^
235 restraints	Δρ_min_ = −0.48 e Å^−^^3^

### Special details

**Table:**

Geometry. All esds (except the esd in the dihedral angle between two l.s. planes) are estimated using the full covariance matrix. The cell esds are taken into account individually in the estimation of esds in distances, angles and torsion angles; correlations between esds in cell parameters are only used when they are defined by crystal symmetry. An approximate (isotropic) treatment of cell esds is used for estimating esds involving l.s. planes.
Refinement. Refinement of *F*^2^ against ALL reflections. The weighted *R*-factor wR and goodness of fit *S* are based on *F*^2^, conventional *R*-factors *R* are based on *F*, with *F* set to zero for negative *F*^2^. The threshold expression of *F*^2^ > σ(*F*^2^) is used only for calculating *R*-factors(gt) etc. and is not relevant to the choice of reflections for refinement. *R*-factors based on *F*^2^ are statistically about twice as large as those based on *F*, and *R*- factors based on ALL data will be even larger.

### Fractional atomic coordinates and isotropic or equivalent isotropic displacement parameters (Å^2^)

**Table:**

	*x*	*y*	*z*	*U*_iso_*/*U*_eq_	Occ. (<1)
N1	0.15275 (14)	0.3564 (5)	0.42609 (17)	0.0414 (6)	
H1A	0.1427	0.4860	0.3832	0.050*	
H1B	0.1304	0.3936	0.4697	0.050*	
H1C	0.1311	0.2092	0.3953	0.050*	
O1	0.49349 (12)	0.2376 (5)	0.60339 (18)	0.0624 (7)	
C1	0.40946 (18)	0.2556 (6)	0.5644 (2)	0.0458 (7)	
C4	0.24232 (16)	0.3242 (5)	0.47587 (19)	0.0366 (6)	
C3	0.29478 (19)	0.4801 (6)	0.4497 (2)	0.0515 (8)	
H3A	0.2739	0.6074	0.4021	0.062*	
C5	0.27196 (18)	0.1368 (7)	0.5450 (2)	0.0497 (8)	
H5A	0.2360	0.0333	0.5625	0.060*	
C6	0.35635 (19)	0.1007 (7)	0.5895 (2)	0.0558 (9)	
H6A	0.3769	−0.0286	0.6364	0.067*	
C2	0.37808 (19)	0.4465 (7)	0.4943 (3)	0.0561 (9)	
H2A	0.4137	0.5527	0.4773	0.067*	
C8	0.6217 (2)	0.0478 (10)	0.6954 (3)	0.0783 (12)	
H8A	0.6483	−0.0935	0.7380	0.117*	
H8B	0.6405	0.2108	0.7279	0.117*	
H8C	0.6347	0.0396	0.6378	0.117*	
C7	0.5304 (2)	0.0266 (8)	0.6678 (3)	0.0632 (10)	
H7A	0.5107	−0.1390	0.6359	0.076*	
H7B	0.5164	0.0372	0.7254	0.076*	
O1W	0.08195 (12)	0.8613 (5)	0.44539 (16)	0.0473 (5)	
H1WB	0.0467 (16)	0.843 (9)	0.3908 (11)	0.086 (15)*	
H1WA	0.071 (2)	0.856 (9)	0.4948 (14)	0.086 (15)*	
P1	0.11668 (4)	0.96937 (15)	0.18642 (5)	0.0396 (3)	
F3	0.0519 (7)	1.103 (3)	0.2240 (8)	0.064 (2)	0.73 (3)
F5	0.1765 (9)	0.833 (3)	0.1430 (10)	0.065 (2)	0.73 (3)
F4	0.0451 (7)	0.782 (2)	0.1246 (9)	0.072 (2)	0.73 (3)
F1	0.0911 (6)	1.1714 (17)	0.0948 (6)	0.0618 (15)	0.73 (3)
F2	0.1410 (8)	0.783 (2)	0.2777 (7)	0.086 (2)	0.73 (3)
F6	0.1869 (6)	1.169 (2)	0.2477 (7)	0.0704 (18)	0.73 (3)
F6'	0.1908 (13)	1.081 (6)	0.2715 (16)	0.066 (4)	0.27 (3)
F4'	0.0396 (15)	0.830 (6)	0.1040 (17)	0.053 (4)	0.27 (3)
F2'	0.1153 (17)	0.730 (5)	0.256 (2)	0.070 (4)	0.27 (3)
F3'	0.0594 (18)	1.160 (7)	0.2229 (19)	0.056 (4)	0.27 (3)
F5'	0.184 (3)	0.796 (8)	0.164 (3)	0.062 (4)	0.27 (3)
F1'	0.1166 (18)	1.168 (5)	0.1154 (19)	0.075 (4)	0.27 (3)

### Atomic displacement parameters (Å^2^)

**Table:**

	*U*^11^	*U*^22^	*U*^33^	*U*^12^	*U*^13^	*U*^23^
N1	0.0383 (13)	0.0429 (13)	0.0428 (13)	−0.0011 (11)	0.0147 (11)	−0.0042 (11)
O1	0.0333 (11)	0.0710 (15)	0.0758 (16)	0.0024 (11)	0.0119 (11)	0.0209 (13)
C1	0.0335 (14)	0.0500 (17)	0.0516 (17)	0.0022 (13)	0.0128 (13)	0.0029 (14)
C4	0.0337 (14)	0.0378 (15)	0.0383 (14)	−0.0002 (12)	0.0132 (12)	−0.0041 (12)
C3	0.0425 (16)	0.0509 (18)	0.0603 (19)	0.0031 (14)	0.0181 (15)	0.0191 (16)
C5	0.0406 (17)	0.0564 (19)	0.0508 (17)	−0.0050 (15)	0.0153 (14)	0.0124 (15)
C6	0.0443 (18)	0.060 (2)	0.0577 (19)	0.0035 (16)	0.0128 (15)	0.0216 (17)
C2	0.0425 (17)	0.0553 (19)	0.073 (2)	−0.0024 (15)	0.0237 (16)	0.0172 (17)
C8	0.045 (2)	0.108 (3)	0.071 (3)	0.018 (2)	0.0099 (19)	0.020 (2)
C7	0.0450 (18)	0.073 (2)	0.064 (2)	0.0094 (18)	0.0113 (16)	0.0147 (19)
O1W	0.0395 (12)	0.0532 (13)	0.0448 (13)	−0.0011 (10)	0.0105 (11)	0.0014 (11)
P1	0.0366 (4)	0.0479 (5)	0.0323 (4)	0.0065 (3)	0.0103 (3)	−0.0011 (3)
F3	0.055 (3)	0.082 (6)	0.065 (3)	0.018 (3)	0.034 (2)	−0.005 (3)
F5	0.055 (3)	0.086 (4)	0.054 (5)	0.022 (2)	0.019 (4)	−0.012 (3)
F4	0.068 (3)	0.074 (4)	0.071 (5)	−0.032 (3)	0.022 (3)	−0.011 (3)
F1	0.054 (4)	0.073 (2)	0.054 (3)	0.014 (3)	0.014 (2)	0.026 (2)
F2	0.092 (6)	0.101 (5)	0.054 (3)	0.027 (4)	0.015 (3)	0.036 (3)
F6	0.057 (2)	0.078 (4)	0.066 (4)	−0.013 (3)	0.011 (2)	−0.025 (3)
F6'	0.042 (4)	0.089 (9)	0.054 (7)	0.011 (6)	0.004 (5)	−0.032 (6)
F4'	0.044 (5)	0.075 (8)	0.040 (6)	−0.004 (5)	0.015 (4)	−0.013 (6)
F2'	0.074 (10)	0.075 (7)	0.060 (8)	0.017 (6)	0.025 (6)	0.029 (6)
F3'	0.048 (6)	0.066 (9)	0.042 (6)	0.022 (6)	0.003 (5)	−0.007 (5)
F5'	0.050 (6)	0.087 (10)	0.049 (10)	0.018 (6)	0.018 (7)	−0.017 (7)
F1'	0.065 (10)	0.081 (6)	0.066 (7)	−0.007 (7)	0.010 (6)	0.035 (6)

### Geometric parameters (Å, °)

**Table:**

N1—C4	1.474 (4)	C8—H8B	0.9600
N1—H1A	0.8900	C8—H8C	0.9600
N1—H1B	0.8900	C7—H7A	0.9700
N1—H1C	0.8900	C7—H7B	0.9700
O1—C1	1.370 (4)	O1W—H1WB	0.821 (10)
O1—C7	1.428 (4)	O1W—H1WA	0.82 (3)
C1—C6	1.373 (4)	P1—F1'	1.46 (2)
C1—C2	1.384 (4)	P1—F6'	1.546 (18)
C4—C5	1.358 (4)	P1—F4	1.575 (10)
C4—C3	1.376 (4)	P1—F5	1.578 (13)
C3—C2	1.371 (4)	P1—F2	1.580 (8)
C3—H3A	0.9300	P1—F3	1.590 (11)
C5—C6	1.389 (4)	P1—F6	1.598 (7)
C5—H5A	0.9300	P1—F5'	1.60 (4)
C6—H6A	0.9300	P1—F2'	1.61 (2)
C2—H2A	0.9300	P1—F4'	1.61 (3)
C8—C7	1.500 (5)	P1—F3'	1.63 (3)
C8—H8A	0.9600	P1—F1	1.632 (7)
			
C4—N1—H1A	109.5	F6'—P1—F3	92.8 (9)
C4—N1—H1B	109.4	F4—P1—F3	87.1 (6)
H1A—N1—H1B	109.5	F5—P1—F3	176.5 (7)
C4—N1—H1C	109.5	F2—P1—F3	87.9 (6)
H1A—N1—H1C	109.5	F1'—P1—F6	76.1 (9)
H1B—N1—H1C	109.5	F4—P1—F6	177.8 (5)
C1—O1—C7	118.8 (3)	F5—P1—F6	91.6 (8)
O1—C1—C6	125.2 (3)	F2—P1—F6	89.7 (3)
O1—C1—C2	115.5 (3)	F3—P1—F6	91.4 (6)
C6—C1—C2	119.4 (3)	F1'—P1—F5'	92.5 (15)
C5—C4—C3	120.9 (3)	F6'—P1—F5'	85.2 (18)
C5—C4—N1	119.7 (3)	F4—P1—F5'	91.5 (18)
C3—C4—N1	119.4 (3)	F2—P1—F5'	81.6 (13)
C2—C3—C4	119.5 (3)	F3—P1—F5'	169.4 (13)
C2—C3—H3A	120.2	F6—P1—F5'	90.3 (18)
C4—C3—H3A	120.2	F1'—P1—F2'	174.5 (15)
C4—C5—C6	119.6 (3)	F6'—P1—F2'	88.1 (9)
C4—C5—H5A	120.2	F4—P1—F2'	73.3 (9)
C6—C5—H5A	120.2	F5—P1—F2'	95.5 (12)
C1—C6—C5	120.1 (3)	F3—P1—F2'	85.4 (12)
C1—C6—H6A	119.9	F6—P1—F2'	108.2 (8)
C5—C6—H6A	119.9	F5'—P1—F2'	84.1 (17)
C3—C2—C1	120.4 (3)	F1'—P1—F4'	89.3 (11)
C3—C2—H2A	119.8	F6'—P1—F4'	174.7 (12)
C1—C2—H2A	119.8	F5—P1—F4'	89.5 (10)
C7—C8—H8A	109.5	F2—P1—F4'	105.3 (9)
C7—C8—H8B	109.5	F3—P1—F4'	87.1 (10)
H8A—C8—H8B	109.5	F6—P1—F4'	164.9 (9)
C7—C8—H8C	109.5	F5'—P1—F4'	94.0 (19)
H8A—C8—H8C	109.5	F2'—P1—F4'	86.7 (10)
H8B—C8—H8C	109.5	F1'—P1—F3'	89.0 (15)
O1—C7—C8	107.4 (3)	F6'—P1—F3'	86.7 (13)
O1—C7—H7A	110.2	F4—P1—F3'	96.0 (12)
C8—C7—H7A	110.2	F5—P1—F3'	169.1 (14)
O1—C7—H7B	110.2	F2—P1—F3'	94.9 (12)
C8—C7—H7B	110.2	F6—P1—F3'	82.3 (12)
H7A—C7—H7B	108.5	F5'—P1—F3'	171.8 (19)
H1WB—O1W—H1WA	122 (2)	F2'—P1—F3'	94.9 (17)
F1'—P1—F6'	96.0 (11)	F4'—P1—F3'	94.1 (14)
F1'—P1—F4	102.6 (9)	F6'—P1—F1	109.3 (11)
F6'—P1—F4	161.3 (10)	F4—P1—F1	89.4 (4)
F1'—P1—F5	80.8 (12)	F5—P1—F1	87.8 (5)
F6'—P1—F5	90.7 (10)	F2—P1—F1	177.8 (5)
F4—P1—F5	89.9 (8)	F3—P1—F1	90.3 (5)
F1'—P1—F2	164.6 (8)	F6—P1—F1	89.0 (3)
F6'—P1—F2	69.4 (10)	F5'—P1—F1	100.2 (13)
F4—P1—F2	91.9 (4)	F2'—P1—F1	162.3 (8)
F5—P1—F2	94.0 (6)	F4'—P1—F1	75.9 (10)
F1'—P1—F3	98.1 (12)	F3'—P1—F1	83.1 (11)
			
C7—O1—C1—C6	6.9 (5)	C2—C1—C6—C5	−0.3 (5)
C7—O1—C1—C2	−172.9 (3)	C4—C5—C6—C1	0.7 (5)
C5—C4—C3—C2	−0.2 (5)	C4—C3—C2—C1	0.7 (5)
N1—C4—C3—C2	−178.5 (3)	O1—C1—C2—C3	179.3 (3)
C3—C4—C5—C6	−0.5 (5)	C6—C1—C2—C3	−0.4 (5)
N1—C4—C5—C6	177.8 (3)	C1—O1—C7—C8	177.4 (3)
O1—C1—C6—C5	180.0 (3)		

### Hydrogen-bond geometry (Å, °)

**Table:**

Cg1 is the centroid of the C1–C6 ring.

**Table:**

*D*—H···*A*	*D*—H	H···*A*	*D*···*A*	*D*—H···*A*
N1—H1A···F2'	0.89	2.16	3.03 (2)	167
N1—H1A···F2	0.89	2.17	3.049 (10)	169
N1—H1B···F1^i^	0.89	2.22	3.066 (8)	158
N1—H1B···F1'^i^	0.89	2.28	3.09 (3)	152
N1—H1C···O1W^ii^	0.89	2.22	2.883 (3)	131
N1—H1C···F3^ii^	0.89	2.46	3.137 (12)	133
N1—H1C···F3'^ii^	0.89	2.40	3.01 (3)	126
N1—H1C···F6'^ii^	0.89	2.51	2.96 (2)	112
O1W—H1WB···F3'^iii^	0.82 (1)	2.20 (3)	2.97 (3)	156 (4)
O1W—H1WB···F3^iii^	0.82 (1)	2.28 (3)	3.030 (12)	152 (4)
O1W—H1WA···F4^i^	0.82 (3)	2.24 (2)	3.040 (12)	164 (4)
O1W—H1WA···F4'^i^	0.82 (3)	2.11 (3)	2.88 (2)	155 (4)
O1W—H1WA···F5^i^	0.82 (3)	2.48 (4)	2.951 (16)	117 (3)
C8—H8C···Cg1^iv^	0.96	3.16	4.023 (5)	150

Symmetry codes: (i) *x*, −*y*+3/2, *z*+1/2; (ii) *x*, *y*−1, *z*; (iii) −*x*, *y*−1/2, −*z*+1/2; (iv) −*x*+1, −*y*, −*z*+1.
